# Grandmaternal allergen sensitization reprograms epigenetic and airway responses to allergen in second-generation offspring

**DOI:** 10.1152/ajplung.00103.2023

**Published:** 2023-10-10

**Authors:** Katie M. Lebold, Madeline Cook, Alexandra B. Pincus, Kimberly A. Nevonen, Brett A. Davis, Lucia Carbone, Gina N. Calco, Aubrey B. Pierce, Becky J. Proskocil, Allison D. Fryer, David B. Jacoby, Matthew G. Drake

**Affiliations:** ^1^Department of Emergency Medicine, Stanford University School of Medicine, Palo Alto, California, United States; ^2^Division of Pulmonary, Allergy and Critical Care Medicine, Department of Medicine, Oregon Health and Science University, Portland, Oregon, United States; ^3^Knight Cardiovascular Institute Epigenetics Consortium, Oregon Health and Science University, Portland, Oregon, United States; ^4^Department of Medicine, Oregon Health and Science University, Portland, Oregon, United States; ^5^Department of Molecular and Medical Genetics, Oregon Health and Science University, Portland, Oregon, United States; ^6^Department of Medical Informatics and Clinical Epidemiology, Oregon Health and Science University, Portland, Oregon, United States

**Keywords:** airway hyperreactivity, allergen, asthma, epigenetics, maternal asthma

## Abstract

Asthma susceptibility is influenced by environmental, genetic, and epigenetic factors. DNA methylation is one form of epigenetic modification that regulates gene expression and is both inherited and modified by environmental exposures throughout life. Prenatal development is a particularly vulnerable time period during which exposure to maternal asthma increases asthma risk in offspring. How maternal asthma affects DNA methylation in offspring and what the consequences of differential methylation are in subsequent generations are not fully known. In this study, we tested the effects of grandmaternal house dust mite (HDM) allergen sensitization during pregnancy on airway physiology and inflammation in HDM-sensitized and challenged second-generation mice. We also tested the effects of grandmaternal HDM sensitization on tissue-specific DNA methylation in allergen-naïve and -sensitized second-generation mice. Descendants of both allergen- and vehicle-exposed grandmaternal founders exhibited airway hyperreactivity after HDM sensitization. However, grandmaternal allergen sensitization significantly potentiated airway hyperreactivity and altered the epigenomic trajectory in second-generation offspring after HDM sensitization compared with HDM-sensitized offspring from vehicle-exposed founders. As a result, biological processes and signaling pathways associated with epigenetic modifications were distinct between lineages. A targeted analysis of pathway-associated gene expression found that Smad3 was significantly dysregulated as a result of grandmaternal allergen sensitization. These data show that grandmaternal allergen exposure during pregnancy establishes a unique epigenetic trajectory that reprograms allergen responses in second-generation offspring and may contribute to asthma risk.

**NEW & NOTEWORTHY** Asthma susceptibility is influenced by environmental, genetic, and epigenetic factors. This study shows that maternal allergen exposure during pregnancy promotes unique epigenetic trajectories in second-generation offspring at baseline and in response to allergen sensitization, which is associated with the potentiation of airway hyperreactivity. These effects are one mechanism by which maternal asthma may influence the inheritance of asthma risk.

## Graphical abstract

**Figure:**
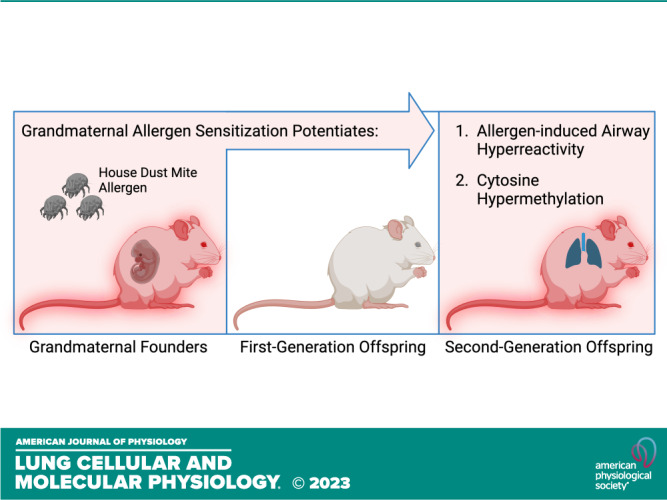

## INTRODUCTION

Asthma is a chronic inflammatory airway disease that is characterized by airflow obstruction and airway hyperreactivity (1). Asthma is more common within families (2) due in part to genetic predisposition (3). However, maternal asthma increases childhood asthma risk more than paternal asthma (4) and better asthma control during pregnancy reduces a child’s asthma risk (5), suggesting maternal factors in utero uniquely contribute to a child’s risk of developing asthma.

Cytosine nucleotide methylation/demethylation represents one form of epigenetic modification by which environmental exposures influence gene expression to increase asthma risk (6–9). Studies have found that differential methylation in early childhood predicts later development of asthma (10, 11) and children with asthma exhibit differential genome-wide methylation compared with children without asthma (12–14). The fetal epigenome is particularly susceptible to modification during pregnancy when exposed to maternal factors such as elevated cytokines (15), smoking (16), stress (17), and asthma (18). Postnatal exposures to aeroallergens, air pollutants, and virus infections are also linked to differential DNA methylation (19–21), complicating the relationship between epigenetic signatures at birth and those found in established diseases.

In this study, we tested whether grandmaternal (F0) allergen sensitization during pregnancy influences the epigenetic signature, airway physiology, and inflammatory response of second-generation offspring at baseline and their subsequent response to allergen sensitization in later life. Since epigenetic regulation is cell specific (22), genome-wide cytosine methylation was specifically tested in airway epithelial cells and airway sensory neurons given their respective roles in initiating immunologic responses to aeroallergens and control of bronchoconstriction (23–26).

## METHODS

### Mice

Male and female C57Bl/6J mice (Jackson Laboratories, Bar Harbor, ME) were 8–14 wk old at the time of analysis. Animals were treated in accordance with the United States Animal Welfare Act. The Institutional Animal Care and Use Committee approved all protocols.

### Grandmaternal Allergen Sensitization

**Figure 1.**
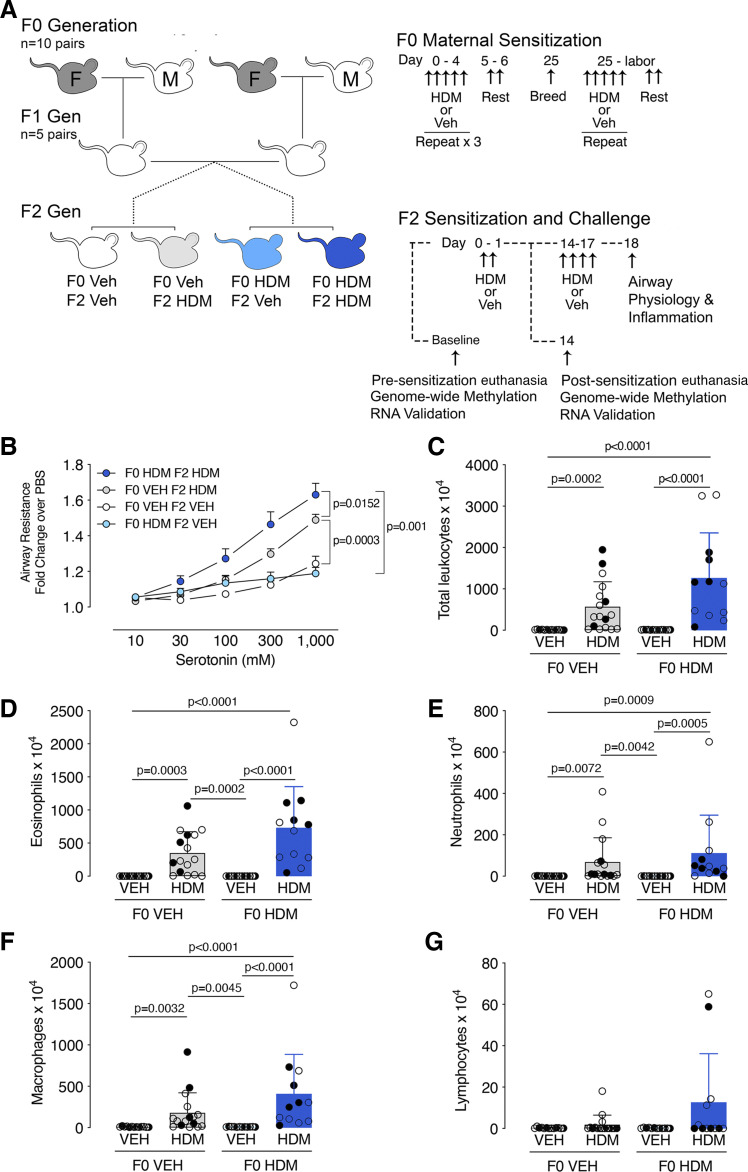
Allergen-induced airway hyperreactivity and inflammation is potentiated in second-generation offspring of house dust mite (HDM)-exposed founders. *A*: female F0 mice (gray mice, “F”) were exposed to intranasal house dust mite (HDM) or vehicle (Veh) for five consecutive days followed by two days of rest for 4 wk, mated, and then exposed to HDM or vehicle 5 days per week through gestation until birth. Male F0 mice (white mouse, “M”) received no treatment. Nonlittermate F1 male and female offspring from matching F0 maternal treatment groups were randomized into breeding pairs and received no treatments. Male and female F2 offspring were sensitized to HDM or treated with Veh on *days 0* and *1* and then challenged with HDM (F0Veh•F1HDM and F0 HDM•F1HDM) or vehicle (F0Veh•F1Veh and F0HDM•F1 Veh) on *days 14*–*17*. Airway physiology and bronchoalveolar lavage leukocytes were measured on HDM-sensitized and challenged F2 mice (*protocol day 18*). A subset of F2 mice were euthanized before allergen sensitization (F0Veh•F1Base and F0HDM•F1Base) and after HDM sensitization (sensitized both *day 0* and *1*, euthanized *day 14*; F0Veh•F1Sens and F0HDM•F1Sens) for methylation experiments. *B*: F2 airway resistance measured in response to aerosolized serotonin. Airway hyperreactivity was present in both HDM-challenged F2 groups (F0Veh•F2HDM and F0HDM•F2HDM). However, HDM-induced airway hyperreactivity was significantly potentiated in HDM-sensitized F2 mice from HDM-exposed F0 mice (F0HDM•F2HDM) compared with F2 mice from vehicle-exposed F0 mice (F0Veh•F2HDM). F0Veh•F2Veh, *n* = 13, *n* = 6 males and *n* = 7 females. F0Veh•F2HDM *n* = 20, *n* = 6 females, *n* = 14 males. F0HDM•F2Veh *n* = 13, *n* = 6 males and *n* = 7 females. F0HDM•F2HDM *n* = 18, *n* = 10 males and *n* = 8 females. Data analyzed with two-way ANOVA with repeated measures. Total (*C*) and differential (*D*–*G*) inflammatory cells and counts were measured in bronchoalveolar lavage of F2 mice. Data were analyzed using Kruskal–Wallis ANOVA with a Dunn’s post test. Closed and open circles represent females and males, respectively.

Nulliparous females were exposed to 25 μg of house dust mite intranasally (HDM, in 25 μL of PBS, Greer Laboratories, *n* = 10 mothers) or vehicle (PBS, *n* = 10 mothers) for 7 or 8 total weeks (Fig. 1*A*). After *week 4*, females were paired with untreated males for breeding. Maternal treatments ceased on delivery.

### First-Generation Offspring Breeding Scheme

First-generation offspring (F1) male mice were randomly mated with nonlittermate F1 female mice from matching F0 treatment cohorts at 8 wk of age (5 total pairs). Breeding schemes were designed to ensure second-generation offspring (F2) mice were evenly descended from F0 founders. F1 mice were all nonsensitized.

### Second-Generation Offspring HDM Sensitization and Challenge

F2 mice 8–12 wk of age were used for methylation, physiology, and airway inflammation experiments. One cohort of F2 mice was euthanized before allergen sensitization for baseline methylation studies. Remaining F2 mice were sensitized with 50 μg of HDM intranasally or vehicle on *days 0* and *1*. On *day 14*, one cohort of mice underwent methylation analysis (postsensitization cohort) whereas a second cohort underwent HDM challenge (25 μg of HDM or vehicle intranasally on *days 14*–*17*). Physiology and inflammation were measured on *day 18*.

### Assessments of Airway Physiology and Inflammation

F2 airway physiology was measured as previously described (25). Briefly, mice were sedated with ketamine (100 mg/kg ip) and xylazine (10 mg/kg ip), paralyzed with succinylcholine (10 mg/kg ip), tracheotomized, and mechanically ventilated via a 21-gauge catheter. A ventilator delivered 200 µL tidal volumes at 125 breaths/min with a 95% oxygen/5% CO_2_ inspired flow and 2 cmH_2_O of positive end-expiratory pressure. Airway pressure was continuously monitored (AD Instruments) and aerosolized serotonin (10–1,000 mM; AeroNeb, Torrington, CT) was delivered at 2-min intervals. Airway resistance, calculated as the difference between peak inspiratory pressure and plateau pressure during an end-inspiratory pause divided by airflow (Resistance = Ppeak-Pplateau/flow), was measured 60 s after each serotonin dose and expressed as fold change over PBS.

Bronchoalveolar lavage was performed by instilling 0.5 mL of sterile PBS three times into the lungs. Total and differential cells were determined by hemacytometer and Wright staining, respectively.

### DNA Isolation, Extraction, and Purification

Mice were injected with a lethal dose of pentobarbital (150 mg/kg ip) and perfused with sterile PBS. Airway epithelium was isolated from tracheas and primary bronchi after incubation in RPMI containing 0.5% protease and penicillin-streptomycin-amphotericin B at 4°C overnight. Cell viability was confirmed as >95% using trypan blue. Vagal ganglia were isolated and placed in lysis buffer (Qiagen Puregene, Hilden Germany) with 0.5% SDS and 100 μg/mL of proteinase K at 55°C overnight. DNA was extracted using Gentra Puregene Tissue (Qiagen) and purified using gDNA Clean and Concentrator 10 columns (Zymo Research, Irvine, CA). DNA concentration and purity were assessed by UV absorption (Nanodrop One, Thermo Fisher, Waltham, MA), size analysis was performed using the gDNA Tape method (2200 TapeStation, Agilent, Santa Clara, CA), and quantification was performed using PicoGreen fluorescence assay (Sigma, Burlington, MA).

### Reduced-Representation Bisulfite Sequencing Library Generation and Differential Methylation Analysis

Reduced-representation bisulfite sequencing (RRBS) libraries were generated as previously described (27). Briefly, DNA (100–150 ng) from epithelium or sensory neurons underwent digestion for 10 h at 37°C with *MspI* restriction enzyme (New England Biolabs, Ipswich, MA), followed by purification with AMPure XP (Beckman Coulter, Indianapolis, IN). Libraries were generated with a NEXTflex Bisulfite-Seq Kit (Bioo Scientific Corporation, Austin, TX) paired with the NEBNext Methylated Adaptor (New England Biolabs). Bisulfite conversion was performed with an EZ DNA Methylation-Gold Kit (Zymo Research) followed by PCR amplification with NEBNext Multiplex Oligos (New England Biolabs). Libraries were quantified with the Qubit High Sensitivity dsDNA Assay (Life Technologies, Eugene, OR), and multiplexed for sequencing on the Illumina NextSeq 500 with the high-output, 75-bp cycle protocol.

RRBS reads were analyzed for quality with FastQC (v.0.11.5), followed by trimming with TrimGalore (v.0.5.0) with the “—rrbs” parameter specified. Trimmed reads were aligned to the Ensembl mouse reference genome (GRCm38) with Bismark (v.0.19.0) (28) using default parameters. Alignment rates were ∼75%. For differentially methylated cytosines (DMCs) analysis, only CpGs with ≥10× coverage and <99.9th% of the highest coverage CpGs in at least four replicates per group were considered. In total, 514,227 CpGs were used for epithelial DMCs analysis and 541,043 CpGs were used for neuron DMCs analysis. DMCs were identified using a logistic regression model that utilized a χ^2^ test, taking biological replicates into account and considering sex as a covariate in the model to calculate *P* values. *P* values were corrected to *q* values using the SLIM method. A *q* value <0.1 and an absolute methylation percent difference >10% were considered significant. Differentially methylated region (DMR) analysis was performed by comparing nonoverlapping 1,000 bp segments between experimental and reference genomes using logistic regression, as previously described (29). Genes associated with DMCs and DMRs were annotated using Ensembl annotation GRCm38.92 and a custom script that utilized BEDTools (30) and the genomation R library (31). For intergenic DMRs, the closest gene and the distance between the DMC/DMR and Transcription Start Site (TSS) were also annotated. (Complete DMC and DMR list can be found in Supplemental Data Files S1 and S2.)

### Gene Ontology and Pathway Analysis

Gene ontology (GO, release date January 01, 2021) and pathway analysis of significant DMCs and DMRs were performed with Panther (release date July 28, 2020). Hypo- and hypermethylated regions were jointly analyzed using overrepresentation test with Fischer’s exact test and false discovery rate (FDR) correction (FDR < 0.05), with reported fold enrichment of over- and underrepresented pathways compared with the whole mouse genome (which was used as the reference list) (32). Over- and underrepresented biological process GO terms with >5 and <1,000 genes were visualized using Cytoscape Enrichment Map and shared gene clusters with functionally related GO terms were labeled.

### Transcription Factor Binding Site Analysis

Enrichment of transcription factor binding sites within significant DMRs was performed using HOMER Motif Enrichment “findMotifsGenome” script (33). The percentage of target sequences with a given motif was compared with the percentage of background sequences with a given motif. Only sequences with a *q* value <0.05 were considered significant.

### Real-Time RT-PCR

Airway epithelial genes were selected for quantification by real-time RT PCR based on their *1*) identification during pathway analysis and *2*) association with human asthma risk. RNA was purified using an RNeasy kit (Qiagen). cDNA was generated using Superscript II Reverse Transcriptase (Thermo Fisher) and amplified using a Veriti 96-well Thermal Cycler (Applied Biosystems). Primers were synthesized by Integrated DNA Technologies (Coralville, IA), as follows: SMAD3 forward: 5′-GGT GCG AGA AGG CGG TCA AGA-3′; SMAD3 reverse: 5′-ACA GGC GGC AGT AGA TAA CGT G-3′; PXN forward: 5′-ACC AGT ACC CGC AGA GGA A-3′; PXN reverse: 5′-GCA CCG CAA TCT CCT GGT ATG-3′; Ets1 forward: 5′-GCC GTC GAT CTC AAG CCG A-3′; Ets1 reverse: 5′ TTT GGG GAT TCC CAG TCG CT-3′; Cdh3 forward: 5′-TGG AGC CGA GCC AAG TTC TG-3′; Cdh3 reverse: 5′-TTG GTG GCA TCA CCC ACT CT-3′; Cacna1d forward: 5′-TGT AGG AGT GGC TGG GTT GG-3′; Cacna1d reverse: 5′-ACA CCC AGG GCA ATT CAA ATC C-3′.

### Immunohistochemistry and Nerve Quantification

Lungs were blocked overnight at 4°C in Tris-buffered saline containing 1% Triton X-100, 4% normal goat serum, and 5% powdered milk. Airway nerves were labeled using a rat polyclonal antibody for substance P (BD PharMingen) followed by secondary anti-rat 555 antibodies (Life Technologies). Isotype controls were performed concurrently using rat IgG. Tissues were mounted on well slides, covered with a glass coverslip, and sealed with Permount (Thermo Fisher). *Z*-stack images of airway epithelium were obtained using a Zeiss LSM880 confocal microscope with a ×63/0.45 PlanApo objective with a 2-mm working distance (Carl Zeiss).

Nerve models were generated by applying an intensity-based filament over substance P-positive voxels (Imaris software). Nerve length and branch points were quantified within airway epithelium from this model. Three to five *z* stacks per mouse were averaged to generate a single experimental value for each animal.

### Statistics

Airway responsiveness was analyzed with a repeated-measures two-way ANOVA with a Tukey’s post test (Prism Graphpad). Outliers greater than two standard deviations above or below their sex and group mean were excluded. Final *n* values for each group were as follows: F0Veh•F2Veh *n* = 13, F0Veh•F2HDM *n* = 20, F0HDM•F2Veh *n* = 13, F0HDM•F2HDM *n* = 18. Cell counts and delta CT values were analyzed with a Kruskal–Wallis ANOVA and Dunn’s post test. Nerve morphology was analyzed with an unpaired *t* test. Genomics data were assessed using R as described earlier.

## RESULTS

### Grandmaternal Allergen Sensitization during Pregnancy Potentiates Airway Hyperreactivity in F2 Offspring

Airway reactivity to inhaled serotonin was similar at baseline between vehicle-challenged F2 offspring from both F0 HDM-exposed founders (F0HDM•F2Veh) and vehicle-exposed F0 founders (F0Veh•F2Veh) (Fig. 1*B*). HDM challenge increased airway hyperreactivity in F2 mice from both HDM- and vehicle-exposed F0 lineages. However, airway hyperreactivity was significantly potentiated in F2 offspring from HDM-exposed F0 founders (F0HDM•F2HDM) compared with vehicle-exposed F0 founders (F0Veh•F2HDM).

Total and differential airway inflammatory cell counts differed between HDM- and vehicle-challenged F2 mice in both HDM- (F0HDM•F2Veh vs. F0HDM•F2HDM) and vehicle-exposed lineages (F0Veh•F2Veh vs. F0Veh•F2HDM) (Fig. 1, *C–G*). Grandmaternal allergen sensitization did not modify the F2 inflammatory cell response.

### Grandmaternal Allergen Sensitization during Pregnancy Alters DNA Methylation in Allergen-Naïve Second-Generation Offspring

**Figure 2.**
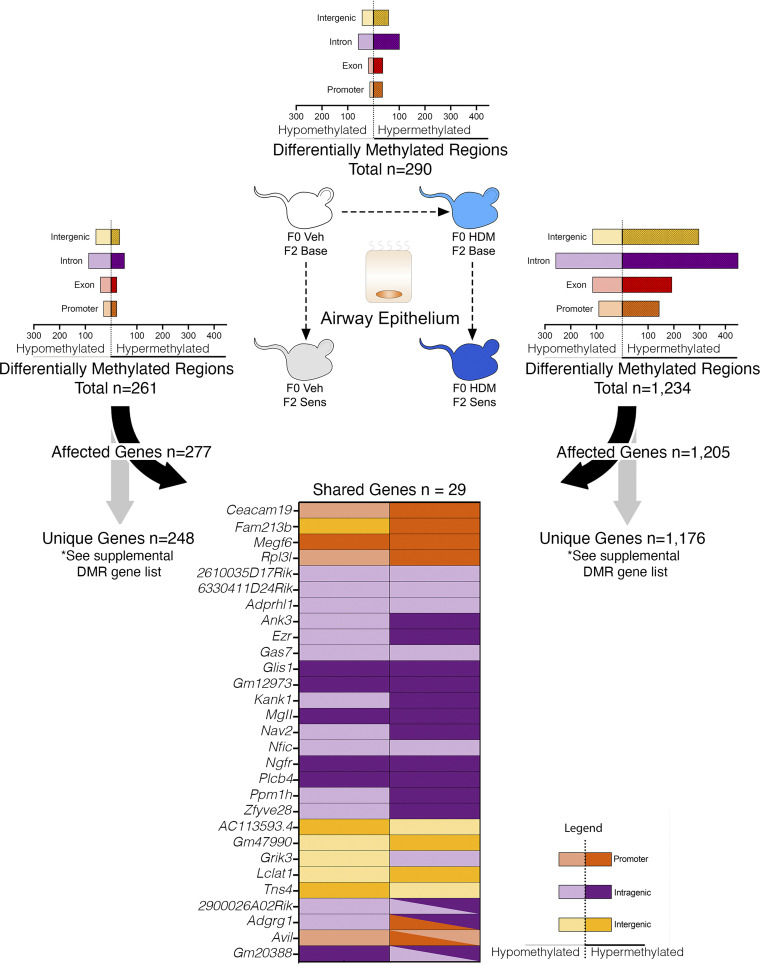
Grandmaternal allergen exposure during pregnancy alters airway epithelial DNA methylation at baseline and after allergen sensitization in second-generation offspring. Whole genome methylation was assessed in airway epithelium of second-generation mice (F2) before and after allergen sensitization. Nonsensitized F2 mice from house dust mite (HDM)-exposed F0 founders had 290 differentially methylated regions (DMRs) (F0 HDM•F2 Base) with a predominantly hypermethylated response compared with control mice (F0 Veh•F2 Base). HDM-sensitized F2 from vehicle-treated F0 founders had 261 DMRs with a predominantly hypomethylated response (F0Veh•F2Sens). In contrast, HDM-sensitized F2 mice from HDM-exposed F0 founders had 1,234 DMRs with a predominantly hypermethylated response (F0 HDM•F2 Sens). In total, only 29 differentially methylated genes were shared between HDM-sensitized F2 mice from HDM-exposed founders compared with HDM-sensitized F2 mice from vehicle-exposed founders. Of these genes, 11 of 29 shared a matching pattern of hyper- or hypomethylation between lineages. *n* = 8 total for each group (*n* = 4 males and *n* = 4 females).

Airway epithelial methylation was compared between F0HDM•F2Base and F0Veh•F2Base mice to determine whether grandmaternal allergen sensitization affects F2 methylation at baseline. In total, F0HDM•F2Base mice contained 290 DMRs (Fig. 2 and Supplemental Data File S1) and 1,390 DMCs (Supplemental Fig. S1 and Supplemental Data File S2) compared with F0Veh•F2Base. DMRs were mapped to 297 genes, of which 177 (61%) were hypermethylated and 113 (39%) were hypomethylated. Fifty-two DMRs (18%) were mapped to promoters (Fig. 2). No biological processes or pathways were identified based on gene ontology (GO) terms for DMRs (Table 1), whereas 74 biologic processes were enriched for DMCs (Supplemental Fig. S2*A*).

**Table 1.** Pathway analysis of differentially methylated regions and cytosines in airway epithelium and vagal ganglia of second-generation F2 mice

Airway Epithelium Group Comparison			
Panther Pathway	No. of Genes	Fold Enrichment	FDR
F0 HDM•F2Base vs. F0 Veh•F2Base			
None			
F0 Veh•F2Sens vs. F0 Veh•F2Base			
** Beta1/2 adrenergic receptor signaling**	**Cacna1c, Cacna1d, Cacnb4, Gngt2** Adcy7, Cacnb1, Cacnb3, Gnb1, Ryr1	**12.22**	**0.018**
** Oxytocin receptor signaling**	**Cacna1d, Cacnb1, Cacnb4, Gngt2, Plcb4** Cacnb3, Gnb1, Plcd3, Prkce	**11.65**	**0.017**
** 5HT2 type receptor signaling**	**Cacna1c, Cacna1d, Cacnb4, Gngt2, Plcb4** Cacnb1, Cacnb3, Gnb1, Plcd3, Prkce	**10.11**	**0.016**
F0 HDM•F2 Sens vs. F0 HDM•F2 Base			
** Endothelin signaling**	**Adcy1, Adcy4, Adcy5, Ece1, Edn2, Furin, Gna14, Itpr1, Itpr2, Map2k1, Pik3c3, Pik3r1, Pik3r5, Plcb4, Prkcd, Sec11a** Adcy7, Akt1, Gnaq, Pik3cd, Plcb3, Prkacb, Prkar1b, Prkce	**5.88**	**<0.001**
** Gonadotropin-releasing hormone receptor signaling**	**Adcy1, Bmp7, Camk2b, Crtc1, Gnb1, Insr, Itpr1, Itpr2, Ksr1, Map2k1, Nfatc2, Pbx1, Pik3r1, Pitx1, Prkcd, Ppp3ca, Pxn, Smad3, Sp1** Akt1, Cacna1c, Cacna1d, Gata4, Gnaq, Hras, Hspa1a, Inhbb, Lhx2, Map2k6, Map3k8, Nfatc4, Otx1, Pcp4, Ppp3ca, Prkag2, Prkar1b, Prkce, Smad2, Tgfb1	**2.43**	**0.029**
** Platelet-derived growth factor signaling**	**Arhgap26, Arhgap27, Elf4, Ephb2, Erg, Ets1, Gm42906, Itpr1, Itpr2, Map2k1, Pik3c3, Pik3r1, Pik3r5, Rps6ka1, Vav2**	**3.16**	**0.008**
** Wnt signaling**	**Arid1b, Axin2, Bcl9, Cdh13, Csnk1g3, Dvl1, Fzd5, Fzd10, Gna14, Gnb1, Itpr1, Itpr2, Lrp5, Nfatc2, Nkd1, Pcdh1, Pcdhgb2, Plcb4, Ppp2r5c, Ppp3ca, Prkcd, Sag, Smarcd1, Tle3, Wnt3a** Acta1, Acte1, Ankrd6, Cdh2, Cdh3, Cdh23, Ctbp1, Fat2, Fzd1, Fzd7, Gm37388, Gnaq, Gng7, Kremen1, Lef1, Myh6, Nfatc4, Pcdhga10, Plcb3, Ppard, Prkce, Tle4, Wnt4, Wnt5a, Wnt5b, Wnt10b	**2.52**	**0.004**

Vagal Ganglia Group Comparison			
Panther Pathway	Gene Name	Fold Enrichment	FDR
F0 HDM•F2Base vs. F0 Veh•F2Base			
None			
F0 Veh•F2Sens vs. F0 Veh•F2Base			
** Metabotropic glutamate receptor group III**	**Grik3, Grik4, Grm6, Prkar1b, Prkar2b**	**7.58**	**0.038**
** Platelet derived growth factor signaling**	**Arhgap26, Arhgap27, Fli1, Gab1, Grap2, Prr5** Arhgap15, Elf4, Ephb2, Gm49337, Itpr1, Itpr3, Map2k2, Mtor, Rasal1, Rps6ka3, Spdef, Stat3, Vav1	**5.05**	**0.053**
F0 HDM•F2Sens vs. F0 HDM•F2Base			
None			

Pathways and genes enriched for both differentially methylated regions (DMRs) and differentially methylated cytosines (DMCs) are denoted in bold font. All pathways identified in DMR analysis were similarly identified in DMC analysis. Additional genes identified on DMC analysis alone are listed in normal font.

### F2 Allergen Sensitization Alone Provokes a Predominantly Hypomethylated Response in Airway Epithelium of Offspring from Control Founders

F0Veh•F2Sens mice were compared with F0Veh•F2Base mice to test whether F2 allergen sensitization alone affects epithelial methylation. In total, 261 DMRs (Fig. 2) and 1,362 DMCs (Supplemental Fig. S1) were identified. For DMRs, 161 (61.7%) were hypomethylated, 100 (38.3%) were hypermethylated, and 20.3% were mapped to promoters.

DMR- and DMC-associated pathways included β-1 and β-2 adrenergic signaling, oxytocin receptor signaling, and 5HT2 serotonin receptor signaling pathways (Table 1 and Supplemental Table S1). Enriched biological processes included cell growth and migration, actin organization, and neuron development (Supplemental Fig. S2*B*).

### Grandmaternal Allergen Sensitization during Pregnancy Potentiates DNA Methylation and Alters Enriched Pathways in Allergen-Sensitized F2 Offspring

The number of DMRs and DMCs was significantly increased in the airway epithelium of allergen-sensitized F2 offspring from HDM-exposed founders (F0HDM•F2Sens) compared with nonsensitized F2 offspring from HDM founders (F0HDM•F2Base). In total, 1,234 DMRs (Fig. 2) and 3,756 DMCs (Supplemental Fig. S1) were identified. Grandmaternal allergen promoted a predominantly hypermethylated response in F0HDM•F2Sens offspring, which included 809 (65.6%) hypermethylated DMRs with only 425 (34.4%) hypomethylated DMRs. Two hundred thirty-six (19.1%) DMRs were mapped to promoters (Fig. 2).

DMR-associated pathways in F0HDM•F2Sens offspring included endothelin, platelet-derived growth factor (PDGF), Wnt, and gonadotropin-releasing hormone signaling pathways (Table 1). GO terms for DMCs were mapped to 418 biological processes after allergen sensitization in F0HDM•F2Sens offspring compared with 97 after allergen sensitization in F0Veh•F2Sens (Supplemental Fig. S2*C*), including axonogenesis, cell proliferation, innate immunity, cytoskeleton regulation, and kinase activity regulation.

### Epigenetic Changes in Airway Epithelial Cells after F2 Allergen Sensitization Are Uniquely Regulated by Grandmaternal Allergen Sensitization

Only 29 genes were shared among the 1,205 DMR-associated genes in F0HDM•F2Sens mice and 277 DMR-associated genes in F0Veh•F2Sens mice (Fig. 2). Of these, only 11 genes had a matching methylation pattern, indicating that F0 allergen exposure provokes a fundamentally unique F2 methylation response after allergen sensitization.

### Multiple Transcription Factor Binding Sites Are Enriched in Allergen-Sensitized Offspring from Allergen-Exposed Founders

Enriched transcription factor binding sites in epithelial DMRs of allergen-sensitized F2 mice from allergen-exposed founders (F0HDM•F2Sens vs. F0HDM•F2Base) included AP-1 and p53 family factors (Supplemental Table S2).

### Pathway-Associated Gene Expression Is Altered by Grandmaternal Allergen Sensitization during Pregnancy

Smad3 expression was significantly upregulated in airway epithelium of F2 offspring from allergen-exposed F0 founders (F0HDM•F2Base and F0HDM•F2Sens) (Supplemental Fig. S3). Differential expression of paxillin was also observed between HDM-sensitized and nonsensitized F2 offspring from allergen-exposed F0 founders, although this difference did not reach statistical significance (*P* = 0.06). Expression was similar for other selected genes.

### Grandmaternal Allergen Sensitization Influences Epigenetic Signatures of Vagal Ganglion Sensory Neurons

**Figure 3.**
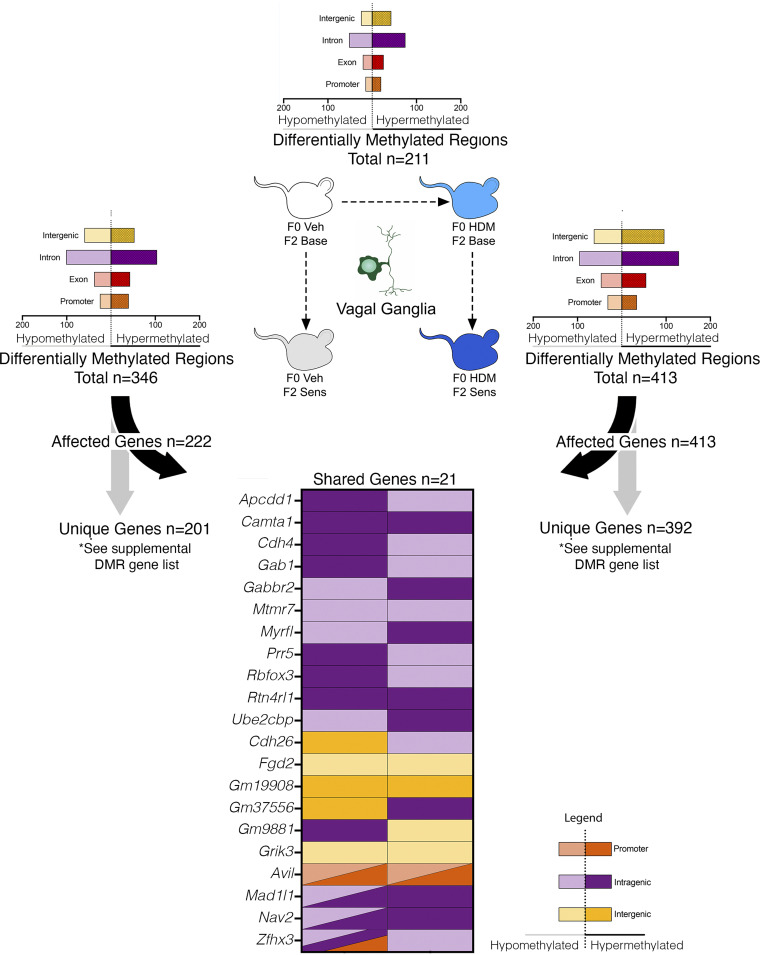
Grandmaternal allergen exposure during pregnancy alters vagal ganglion DNA methylation at baseline and after allergen sensitization in second-generation offspring. Grandmaternal house dust mite (HDM) sensitization resulted in 211 differentially methylated regions (DMRs) (F0 HDM•F2 Base vs. F0 Veh•F2 Base) in vagal ganglia of F2 offspring at baseline. F2 HDM sensitization alone resulted in 346 DMRs (F0Veh•F2Sens vs. F0 Veh•F2 Base). In contrast, HDM sensitization of F2 mice from HDM-exposed founders resulted in 413 DMRs (F0 HDM•F2 Sens vs. F0 HDM•F2 Base). Only 21 genes were shared between F0 Veh•F2 Sens and F0 HDM•F2 Sens groups. Of these 21 genes, only seven had a matching pattern of hyper- or hypomethylation between lineages. *n* = 8 total for each group (*n* = 4 males and *n* = 4 females).

A total of 211 DMRs and 901 DMCs were detected in vagal ganglia from F0HDM•F2Base mice, including 127 (60.2%) hypermethylated DMRs and 536 (59.5%) hypermethylated DMCs (Fig. 3 and Supplemental Fig. S4). The 211 DMRs corresponded to 222 unique genes, of which only 15 were similarly detected in airway epithelium (Supplemental Fig. S5), indicating that epigenetic changes occur in a tissue-specific context.

For F0Veh•F2Sens, a total of 346 DMRs and 1,467 DMCs were detected (Fig. 3 and Supplemental Fig. S4), representing 174 (50.3%) hypermethylated DMRs and 795 (54.2%) hypermethylated DMCs. Associated pathways included metabotropic glutamate receptor group III, platelet-derived growth factor and cholecystokinin receptor signaling (Table 1). Enriched biological processes included axonogenesis, cell projection, cell adhesion, and synapse organization among others (Supplemental Fig. S6).

Four hundred and thirteen DMRs and 1,787 DMCs were identified in vagal ganglia from F0HDM•F2Sens mice (Fig. 3 and Supplemental Fig. S4), of which 242 (58.6%) DMRs and 892 (49.9%) DMCs were hypermethylated. No DMR-associated pathways were detected (Supplemental Table S3). Enriched biological processes included axonogenesis and transmembrane cation transport (Supplemental Fig. S6).

Comparison of DMR-associated genes in allergen-sensitized F2 offspring from HDM-exposed and vehicle-exposed F0 founders (F0HDM•F2Sens vs. F0Veh•F2Sens) identified only 21 shared genes out of 222 total genes in F0Veh•F2Sens mice and 413 total genes in F0HDM•F2Sens mice (Fig. 3).

### Few Transcription Factor Binding Sites Were Enriched in DMRs of Vagal Ganglia

Only the ETS family member Elk4 transcription factor binding site was enriched in vagal neuron DMRs from HDM-sensitized offspring from both allergen-exposed and vehicle-exposed founders (F0HDM•F2Sens vs. F0HDM•F2Base and F0Veh•F2Sens vs. F0Veh•F2Base) (Supplemental Table S2).

### Airway Sensory Nerve Density Is Unaffected by Grandmaternal Allergen Sensitization

Previously, we reported that maternal HDM sensitization and maternal interleukin (IL)-5 increase airway sensory nerve density in first-generation offspring (25, 26). However, sensory nerve density, represented as total nerve length, and nerve complexity, represented by the number of branch points, were similar between F2 offspring from HDM-exposed and vehicle-exposed grandmaternal founders (Supplemental Fig. S7), indicating that maternal HDM’s effects on airway sensory nerve density are isolated to first-generation offspring.

## DISCUSSION

DNA methylation represents one epigenetic mechanism by which rapid phenotypic adaptations resulting from environmental exposures can be transmitted to future generations. Exposures to maternal factors during fetal lung development may be particularly impactful to asthma risk. However, linking specific maternal factors to epigenetic changes and developmental outcomes in humans has been challenging due to human longevity and confounding from a myriad of pre- and postnatal exposures. Maternal smoking has been most strongly linked with epigenetic changes associated with asthma (34–36), as have maternal diet (37), stress (38), and diesel exhaust exposure (39). Individual CpG methylation sites have also been associated with atopy and asthma in children (35, 40). Epigenetic changes influence immune cell development, including Th1, Th2, and T-regulatory cell differentiation (41–43), and differences in immune function can be detected at birth (44, 45), suggesting prenatal programming influences a child’s response to environmental exposures from the earliest moments of life.

In our model, we show that grandmaternal allergen sensitization during pregnancy potentiates allergen-induced airway hyperreactivity in F2 offspring that is associated with significant tissue-specific alterations in offspring’s epigenetic signature at baseline, and fundamentally alters their epigenetic response to allergen sensitization later in life.

DMRs and DMCs in allergen-sensitized F2 offspring from allergen-exposed founders mapped to signaling pathways that regulate lung development and inflammation, including WNT signaling, platelet-derived growth factor signaling, gonadotropin-releasing hormone receptor pathway, and endothelin receptor signaling. WNT proteins in our model included LRP5 and Axin2, which regulate airway branching and alveolar morphogenesis in fetal lungs (46–48), and Nfatc2 (49, 50) and PCDH1 (51–53), which are novel susceptibility loci for allergy and bronchial hyperreactivity in humans, respectively. Expression of *WNT3*, *WNT5a*, *WNT10*, and their receptor, Fzd-5, positively correlated with type 2-high asthma in adults whereas expression of WNT5b negatively correlated with type 2 inflammation (54). Tle3-regulated T cell development (55) and loss of WNT10b enhanced airway inflammation in a murine model (56). Functionally, WNT signaling is critical to epithelial-mesenchymal differentiation during development (57, 58) and at times of lung injury (59), where their ability to broadly influence transcriptional events regulates airway inflammation and epithelial repair.

PDGF family proteins identified in our study are involved in cell proliferation and migration during embryogenesis and contribute to airway remodeling by inducing epithelial metaplasia and airway smooth muscle migration in later life (60). MEK1 and Pi3k mediated epithelial cytokine production after HDM exposure in adult mice (61). Similarly, endothelin pathway mediators played an important role in initiating eosinophilic inflammation after allergen (62), and polymorphisms in the endothelin signaling protein ITPR2 are associated with asthma in humans (63). Thus, differential methylation in our study has identified pathways involving airway immune responses, lung development, and airway contractility.

Several transcription factor binding sites were enriched in epithelial DMRs of allergen-sensitized F2 offspring from allergen-exposed founders, including members of the AP-1 and p53 factor families. AP-1 consists of Fos (c-Fos, FosB, FosL1, FosL2) and Jun (c-Jun, JunB, JunD) proteins, which form dimers that regulate transcription of inflammatory genes relevant to asthma, such as c-Fos and interleukin (IL)-5, and Fra2 and IL-13 (64–66). AP-1 binding also reduces glucocorticoid receptor binding site availability by altering chromatin structure (67, 68), suggesting AP-1 has a role in development of corticosteroid-resistant disease. Accordingly, in asthma and particularly in corticosteroid-resistant asthma, expression of AP-1 dimers (e.g., c-Fos) is increased (69, 70). AP-1 can also be activated by inflammatory cytokines TNF-α and IL-1β, suggesting transcription factor activity is amplified by its own downstream products.

Prior animal studies have similarly shown that maternal exposures mediate transgenerational epigenetic transmission. For example, maternal diesel exhaust altered dendritic cell DNA methylation that persisted across three generations (71). In a second study by Pulczinski et al. (72), maternal HDM exposure altered methylation patterns in whole lung homogenates that were inherited by three successive generations and were associated with enhanced airway inflammation and airway reactivity after HDM challenge. In contrast to Pulczinski et al., our analysis took a cell-specific approach since methylation changes are known to be highly cell specific (further confirmed by significant methylation differences between cell types in our study). The central role of airway epithelium in allergic asthma as both the site of contact for inhaled particles and as a key source of proinflammatory cytokines (73), and the role of airway sensory neurons in the regulation of reflex bronchoconstriction and hyperreactivity, make these two tissues of special interest. In the study by Pulczinski et al. (72), methylation was measured in males after successive HDM challenges in every generation beginning in F1, whereas our study focused specifically on the effects of grandmaternal HDM sensitization on the F2 generation. Consequently, our F1 generation was nonsensitized and methylation changes in F2 mice were measured before HDM sensitization, as well as after HDM sensitization, to enable comparisons between the effects of grandmaternal allergen on F2 at baseline, and after F2 allergen sensitization.

Analysis of a selected subset of differentially methylated pathway-associated genes in airway epithelium found that two genes, Smad3 and Paxillin, were differentially expressed in our cohorts. In human studies, Smad3 hypermethylation was the strongest predictor of childhood asthma in children born to mothers with asthma in three birth cohorts (74). Genome-wide association studies similarly noted an association between single-nucleotide polymorphisms in the Smad3 gene and asthma risk (75, 76). Smad3 activation by TGF-β provokes both airway smooth muscle hyperreactivity (77) and regulates T regulatory cell differentiation (76), suggesting Smad3’s dysregulation in our study may contribute to potentiation of airway hyperreactivity. Paxillin also promotes airway hyperreactivity via its role in the development of airway smooth muscle hypertrophy and by serving as a structural focal adhesion protein on airway epithelium that contributes to barrier integrity (78, 79).

Our analysis included an evaluation of methylation in airway sensory neurons since *1*) airway nerves control bronchoconstriction (80); *2*) sensory nerve remodeling correlated with worse lung function and increased irritant sensitivity in humans with eosinophilic asthma (24); and *3*) exposure to maternal type-2 inflammation increased airway sensory nerve density and nerve-mediated airway hyperreactivity after allergen exposure in first generation mice (25, 26). In the present study, maternal allergen exposure did not affect airway sensory nerve density in F2 mice despite significantly altering sensory nerve methylation at baseline and after allergen sensitization. In total, only 21 genes corresponding to DMRs were shared between allergen-sensitized offspring from allergen-exposed versus control lineages, providing further evidence that grandmaternal allergen sensitization during pregnancy results in substantial tissue-specific epigenetic reprogramming.

Our study has limitations. First, although we show that grandmaternal HDM sensitization during pregnancy creates a tissue-specific epigenetic signature that is established at birth and informs epigenetic trajectories after allergen sensitization, we did not test the mechanisms by which this signature was inherited. We analyzed epigenetic changes in F2 offspring and as such cannot conclude whether the observed epigenetic changes were inherited transgenerationally or reflect direct effects on in utero gamete development (i.e., grandmaternal HDM exposure continued throughout F2 gamete development in F1 embryos). The same limitation applies to most human asthma epigenetic studies published to date. We also cannot exclude the influence of a dose effect in HDM-sensitized F2 mice from HDM-sensitized F0 founders. However, HDM allergen does not cross the placenta. Thus, any effects from cumulative HDM exposure would reflect factors related to the maternal allergen response, not the direct effects of allergen on the developing gametes. Finally, our study protocol does not differentiate between the effects of pre-existing maternal allergen sensitization from that of ongoing maternal allergen exposure during pregnancy, since all maternal allergen sensitization started before and continued throughout pregnancy.

In summary, our study indicates that grandmaternal allergen sensitization that was established before and continued throughout pregnancy increases airway reactivity in allergen-sensitized and challenged F2 offspring. Grandmaternal allergen sensitization was also associated with reprogramming of F2 epigenetic response to allergen sensitization. We show that epigenetic trajectories are established before birth in response to prenatal exposures, which subsequently direct epigenetic responses to allergen sensitization and may contribute to asthma risk.

## DATA AVAILABILITY

Data will be made available upon reasonable request.

## SUPPLEMENTAL DATA

10.5281/zenodo.8397632 Supplemental Figs. S1–S7, Supplemental Data Files S1 and S2, and Supplemental Tables S1–S3: https://doi.org/10.5281/zenodo.8397632.

## GRANTS

This work was supported by the National Institutes of Health National Heart, Lung, and Blood Institute (NHLBI) Grants HL155623 (to M.D.), HL121254 (to M.D.), HL144008 (to D.J.), HL124165 (to D.J.), HL132414 (to K.L.), and HL131525 (to A.F.).

## DISCLOSURES

No conflicts of interest, financial or otherwise, are declared by the authors.

## AUTHOR CONTRIBUTIONS

K.M.L., L.C., G.N.C., B.J.P., A.D.F., D.B.J., and M.G.D. conceived and designed research; K.M.L., M.C., A.B.P., K.A.N., B.A.D., L.C., G.N.C., A.B.P., B.J.P., A.D.F., D.B.J., and M.G.D. performed experiments; K.M.L., M.C., A.B.P., K.A.N., B.A.D., L.C., G.N.C., A.B.P., B.J.P., A.D.F., D.B.J., M.G.D. analyzed data; K.M.L., M.C., A.B.P., K.A.N., L.C., G.N.C., A.B.P., B.J.P., A.D.F., D.B.J., and M.G.D. interpreted results of experiments; K.M.L., M.C., G.N.C., B.J.P., A.D.F., D.B.J., M.G.D. prepared figures; K.M.L., M.C., G.N.C., B.J.P., A.D.F., D.B.J., and M.G.D. drafted manuscript; K.M.L., M.C., A.B.P., G.N.C., B.J.P., A.D.F., D.B.J., and M.G.D. edited and revised manuscript; K.M.L., M.C., A.B.P., K.A.N., B.A.D., L.C., G.N.C., A.B.P., B.J.P., A.D.F., D.B.J., and M.G.D. approved final version of manuscript.
